# MUC1 induces acquired chemoresistance by upregulating ABCB1 in EGFR-dependent manner

**DOI:** 10.1038/cddis.2017.378

**Published:** 2017-08-10

**Authors:** Wei Jin, Xiaodong Liao, Yaping Lv, Zhi Pang, Yuming Wang, Quanfu Li, Yahui Liao, Qing Ye, Guoqiang Chen, Kewen Zhao, Lei Huang

**Affiliations:** 1 Key Laboratory of Cell Differentiation and Apoptosis of The Chinese Ministry of Education, Department of Pathophysiology, Shanghai Jiao Tong University School of Medicine, Shanghai, China; 2 Department of Cardiothoracic Surgery, Renji Hospital, Shanghai Jiao Tong University School of Medicine, Shanghai, China

## Abstract

Chemoresistance contributes to cancer relapse and increased mortality in a variety of cancer types, raising a pressing need to better understand the underlying mechanism. MUC1 is abnormally overexpressed in numerous carcinomas and associated with poor prognosis. However, the functional significance of MUC1 in chemoresistance has not been fully elucidated. Here, we showed that MUC1 expression was considerably induced in cells that had acquired chemoresistance at both transcriptional and post-translational levels. Using gain- and loss-of function approaches, we demonstrated a critical role of MUC1 in induction of drug resistance. Through stimulation of EGFR activation and nuclear translocation, MUC1 increased the expression of ATP-binding cassette transporter B1 (ABCB1). Remarkably, targeted suppression of EGFR or ABCB1 by both shRNAs and inhibitors effectively reversed chemoresistance. Moreover, co-administration of the inhibitors of MUC1–EGFR–ABCB1 with paclitaxel significantly blocked not only tumor growth but also relapse in xenograft mouse model. Our data collectively support a model in which MUC1 induces acquired chemotherapy resistance by upregulating ABCB1 in an EGFR-dependent manner, providing a novel molecular basis of using the EGFR inhibitor in MUC1-positive cancers to prevent chemotherapy resistance.

Chemoresistance is one of the important mechanisms responsible for tumor recurrence and poor prognosis in a variety of cancer types.^1, 2, 3^ Paclitaxel (PTX) is a tubulin-disrupting drug in the management of a wide range of tumors.^4, 5, 6^ Although studies have uncovered the mechanisms of PTX resistance in several malignancies, many critical issues remain, warranting further investigation. ATP-binding cassette (ABC) transporters are shown to selectively pump out cytotoxic drugs from cells resulting in multidrug resistance.^7^ The human ABC transporter B1 (ABCB1), also known as p-glycoprotein (Pgp), is one of the well-characterized ABC transporters with the broadest substrate specificity. Many chemotherapy drugs for cancer are substrates for ABCB1, including PTX, vincristine, doxorubicin and etoposide.^8, 9^ ABCB1 is found overexpressed in cancer patients with poor response to chemotherapy.^10, 11, 12^ To overcome ABCB1-induced chemoresistance, several pharmacological inhibitors have been developed but with limited success in clinic because of toxicities, which is primarily attributed to the critical functions of ABC transporters in various normal tissues in the physiological clearance of catabolites and xenobiotics.^13, 14^

Mucin 1 (MUC1) is a transmembrane glycoprotein. In normal tissues, MUC1 distributes on the apical surface of luminal epithelial cells and forms a mucinous gel with other mucin members to protect the underlying epithelia.^15, 16^ However, MUC1 is aberrantly glycosylated and overexpressed in many carcinomas and associated with poor outcomes,^17, 18^ including cervical cancer^19^ and lung cancer.^20^ Abundant evidence indicates oncogenic functions for MUC1, which (1) promotes receptor tyrosine kinases activation and downstream signaling^21, 22^ (2) attenuates the apoptotic response to genotoxic and oxidative stress^23^ and regulates the Wnt/*β*-catenin,^24, 25^ p53,^26, 27^ matrix metallopeptidase (MMP13)^28^ and NF-*κ*B^29^ pathways. Moreover, overexpression of MUC1 is found to induce transformation in cells and transgenic mouse models.^15, 18^ Recently, increasing evidence demonstrated a pivotal role of MUC1 in therapeutic resistance in certain tumor types.^30^ MUC1-regulated genes are highly predictive of clinical outcome in breast and lung cancer patients.^20, 31^ Overexpression of MUC1-C induced chemotherapeutic resistance in pancreatic cancer cells by elevating multidrug resistance protein-1 (MRP1, ABCC1).^32^ Our previous studies also demonstrated that MUC1-C associated with ataxia telangiectasia mutated (ATM) and H2AX, protected against IR-induced cell death.^33^

In this study, we aimed at investigating the relationship between MUC1 and PTX resistance, and dissecting the molecular mechanism underlying the chemoresistance. We found that PTX induced MUC1, which then contributed to chemoresistance by upregulation of ABCB1 through cooperation with nuclear EGFR. Our work uncovered a novel mechanism for MUC1-mediated regulation of ABCB1, carrying important therapeutic implication in overcoming the chemoresistance of MUC1-positive tumors.

## Results

### MUC1 expression is induced during acquired chemoresistance

**Figure 1:**
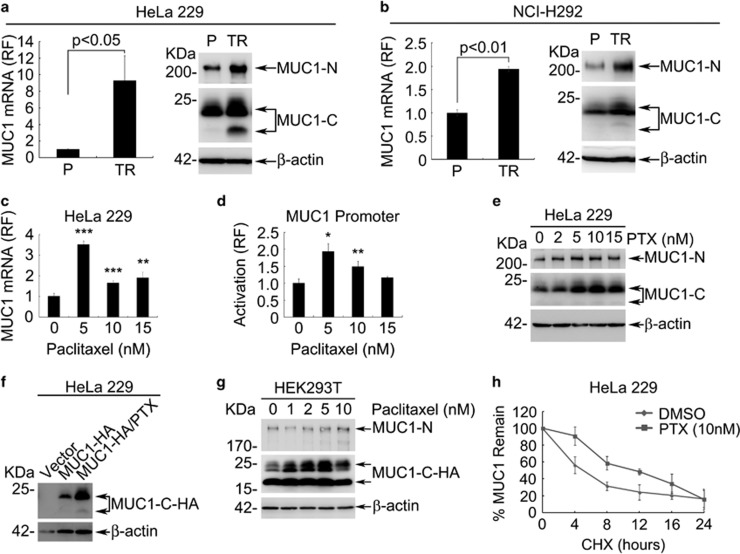
MUC1 expression is induced during acquired chemoresistance. (**a**) The mRNA and protein levels of MUC1 in HeLa229 parent (P) and HeLa229/TR (TR) cells were measured by RT-qPCR (left) and western blot (right). (**b**) The mRNA and protein levels of MUC1 in NCI-H292 parent (P) and PTX-resistant NCI-H292/TR (TR) cells were measured by RT-qPCR (left) and western blot (right). (**c**) HeLa229 cells were treated with different doses of PTX for 48 h. RT-qPCR was carried out to identify the mRNA of MUC1. (**d**) HeLa229 cells were transfected with pGL3-MUC1 promoter (500 ng) then treated with indicated dose of PTX for 48 h. The relative folds of luciferase activity were calculated against 0 nM PTX treatment (line 1). (**e**) HeLa229 cells were treated with different doses of PTX for 48 h. Western blot was carried out to identify the protein of MUC1. (**f**) HeLa229 cells were transfected with pIRESpuro2-MUC1-HA (MUC1-HA) or vector plasmids. Twenty-four hours after transfection, cells expressing MUC1-HA were treated with DMSO (0 nM) or 10 nM PTX for another 24 h, then western blot was carried out to identify the accumulation of exogenous HA tagged MUC1-C. (**g**) HEK293T cells were transfected with pIRESpuro2-MUC1-HA and stable expression clone was selected. The cells were treated with indicated doses of PTX for 48 h. Western blot was carried out to identify the expression of MUC1. (**h**) HeLa229 cells were treated with DMSO (0 nM) or 10 nM PTX for 48 h, then exposed to cycloheximide (CHX) (50 *μ*g/ml) for indicated time. Western blot was carried out, the remaining level of MUC1-C was quantified by Image Studio Lite, version 3.1 (Li-Cor, Lincoln, NE, USA) and then compared with the initial level (0 h). The half-life curve was the average of three independent experiment. Data are shown of three independent experiments, mean±S.D. (*n*=3)

Analysis of ONCOMINE database revealed an overexpression of MUC1 in cervical cancer (Supplementary Figure S1A) and lung cancer (Supplementary Figure S1B). Given the association of MUC1 with chemoresistance, we made an attempt to investigate a potential involvement of MUC1 in chemoresistance in cervical cancer and pulmonary mucoepidermoid lung carcinoma (PMC). We first established a cervical PTX-resistant cell line HeLa229/TR. Long-term treatment with PTX resulted in a substantial induction of MUC1 expression at the mRNA and protein level in HeLa229/TR cells (Figure 1a), which was accompanied with approximately 15-folds increase of IC50 value over that of HeLa229 parental cells (Supplementary Figure S1C). A similar strategy was also used with PMC cell line NCI-H292. The results showed that increased MUC1 expression by PTX treatment was associated with induction of chemoresistance (Figure 1b and Supplementary Figure S1D).

We next examined the role of MUC1 in modulation of cancer cell response to therapy by monitoring MUC1 expression in HeLa229 parental cells treated with PTX. RT-qPCR revealed that the expression of MUC1 mRNA was significantly induced by PTX (Figure 1c), especially at the dose of 5 nM. The induction of MUC1 by 10 and 15 nM PTX was also readily evident but relatively modest. We further substantiated PTX-induced transcription of MUC1 by performing a MUC1 promoter-based transactivation assay. Transcription activity showed a similar pattern to the induction of mRNA (Figure 1d). The results together indicate that PTX transcriptionally upregulated MUC1 expression. We next examined the effect of PTX on MUC1 at the protein level. Both MUC1-N and MUC1-C protein levels were considerably elevated in a dose-dependent manner in PTX-treated cells (Figure 1e). Time-course experiment revealed that treatment of HeLa229 cells with 5 nM PTX resulted in increase in MUC1 protein as early as 48 h (Supplementary Figure S1E). Interestingly, we found that MUC1 protein began to increase at 5 nM but did not further increase at 15 nM. The data suggested a mechanism in addition to transcriptional regulation in PTX-induced MUC1 level. To test this possibility, HeLa229 cells were transiently transfected with MUC1-HA and were exposed to 10 nM PTX. Western blot with an anti-HA antibody indicated that the exogenously expressed MUC1 was also induced by PTX (Figure 1f). Similar results were also seen in HEK293T cells with stably expressed MUC1-HA (Figure 1g). The results indicated a mechanism of post-transcriptional regulation of MUC1 by PTX. Consistence with this notion, half-life assay showed a longer half-life of MUC1 in PTX treatment cells (~12 h) than the control cells (~5 h) (Figure 1h and Supplementary Figure S1F). The results together suggest that PTX treatment upregulated MUC1 transcriptionally as well as post-translationally.

### MUC1 modulates chemosensitivity in both cancers

**Figure 2:**
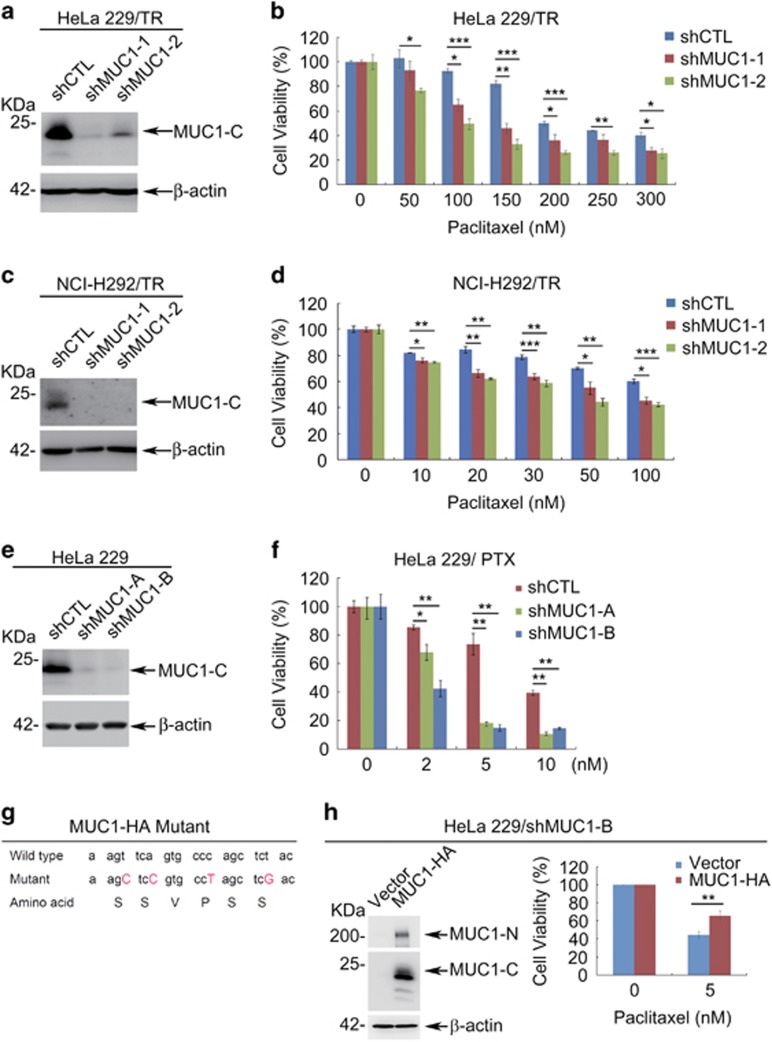
MUC1 modulates chemosensitivity in cancer cells. (**a**) Western blot of indicated proteins in HeLa229/TR/shCTL and HeLa229/TR/shMUC1 cells with *β*-actin as a loading control. (**b**) HeLa229/TR/shCTL and HeLa229/TR/shMUC1s cells were seeded in 96-well plate (6000 cells per well), cultured overnight and then treated with different concentrations of PTX for 48 h. Cell viability was measured by CCK8 assay. (**c**) Western blot of indicated proteins in NCI-H292/TR/shCTL and NCI-H292/TR/shMUC1 cells with *β*-actin as a loading control. (**d**) NCI-H292/TR/shCTL and NCI-H292/TR/shMUC1 cells were seeded in 96-well plate (10000 cells per well), cultured overnight and then treated with different concentrations of PTX for 48 h. Cell viability was measured by CCK8 assay. (**e**) HeLa229 cells were stably transfected with pRNAU6.1-shCTL, pRNAU6.1-shMUC1-A or pRNAU6.1-shMUC1-B plasmids and subjected to western blot with indicated antibodies. (**f**) HeLa229/shCTL and HeLa229/shMUC1 cells were treated with different concentrations of PTX for 48 h. CCK8 assays were applied to detect cell viability. (**g**) A diagram of wild-type and synonymous mutated MUC1 sequences. (**h**) HeLa229/shMUC1-B cells were transfected with shMUC1-B-resistant pIRESpuro2-MUC1-HA (MUC1-HA) or vector plasmids. Western blot was carried out to identify the expression of MUC1 (left). Cells were treated with DMSO (0 nM) or 5 nM of PTX for 48 h. CCK8 assays were applied to detect cell viability (right). Data are shown of three independent experiments, mean±S.D. (*n*=3)

To investigate whether PTX-induced MUC1 was responsible for the observed resistance in cancer cells, we silenced the expression of MUC1 in HeLa229/TR cells. Measurement of the cell response to PTX revealed that the resistance to PTX was significantly diminished upon silencing MUC1 expression in HeLa229/TR (Figures 2a and b and Supplementary Figure S2A), confirming a critical role of MUC1 in the acquired PTX resistance. Knockdown of MUC1 expression in NCI-H292 also indicated an important role of MUC1 in PTX resistance (Figures 2c and d and Supplementary Figure S2B), similar to that in HeLa229. To further substantiate the contribution of MUC1 to the chemosensitivity, we used a different approach to silence MUC1 in HeLa229. Western blot confirmed that the expression of MUC1 was efficiently attenuated in two separate clones expressing the shMUC1-A and shMUC1-B plasmids, respectively (Figure 2e). The results showed that silencing MUC1 expression significantly reduced survival in PTX-treated cells (Figure 2f) and IC50 values for PTX (Supplementary Figure S2C). Of note, silencing MUC1 has little effect on cell viability and proliferation ratio of HeLa229/TR (Supplementary Figure S2D) and HeLa229 cells (Supplementary Figure S2E). We further validated the role of MUC1 by a rescue experiment. Introducing a shMUC1-B-resistant MUC1-HA (Figure 2g) back to HeLa229/shMUC1-B cells restored the resistance to PTX (Figure 2h). These data collectively support that MUC1 has an important role in modulating cancer cell sensitivity to PTX.

### MUC1 elevates ABCB1 upon chemotherapy

**Figure 3:**
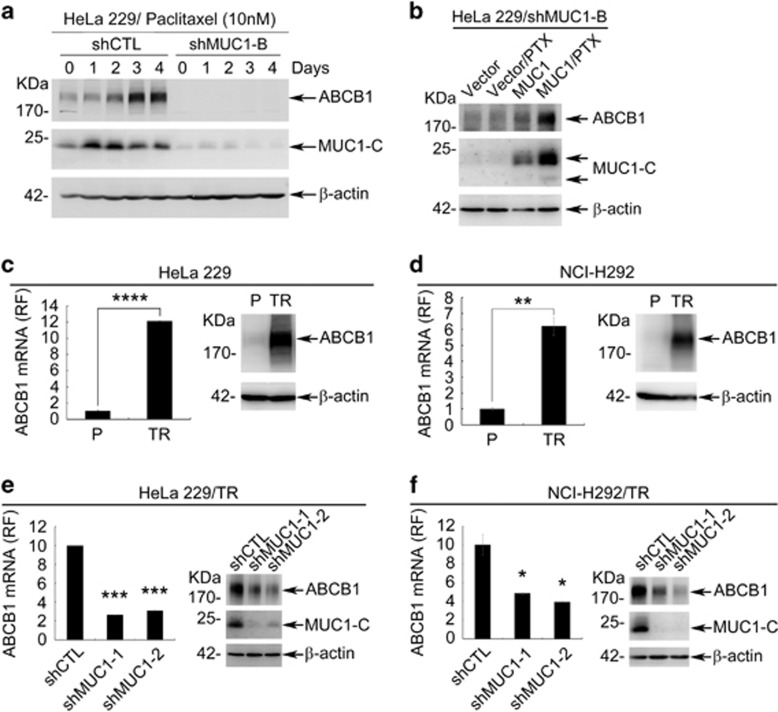
MUC1 upregulates ABCB1 expression upon chemotherapy. (**a**) HeLa229/shCTL and HeLa229/shMUC1-B were treated with 10 nM PTX for indicated time. Western blot was carried out to examine the protein levels of ABCB1 and MUC1-C-terminal (MUC1-C) with *β*-actin as loading control. (**b**) HeLa229/shMUC1-B cells were transfected with shMUC1-B-resistant pIRESpuro2-MUC1-HA (MUC1-HA) or vector plasmids. Twenty-four hours after transfection, cells were treated with PTX (5 nM) for 48 h. Western blot was carried out to identify the expression of ABCB1 protein. (**c** and **d**) The mRNA and protein levels of ABCB1 in HeLa229P/TR (**c**) or NCI-H292P/TR (**d**) cells were detected by RT-qPCR (left) and western blot (right) with *β*-actin as internal control. The PTX resistance cell lines were cultured in medium without PTX. (**e** and **f**) The mRNA and protein levels of ABCB1 in HeLa229/TR/shCTL and HeLa229/TR/shMUC1s (**e**) or NCI-H292/TR/shCTL and NCI-H292/TR/shMUC1s (**f**) cells were detected by RT-qPCR (left) and western blot (right) with *β*-actin as internal control. Data are shown of three independent experiments, mean±S.D. (*n*=3)

It has been widely observed that increased expression of ABC transporters contribute to multidrug resistance in cancer cells.^34^ We thus asked whether ABC transporters may be involved in MUC1-dependent chemoresistance. To this end, we compared MUC1-deficient cells with proficient cells for the expression of ABC transporters. Among nine known transporters associated with PTX resistance, the mRNA (Supplementary Figure S3A) and protein level (Supplementary Figure S3B) of ABCB1 were significantly downregulated in HeLa229/shMUC1s when compared with HeLa229/shCTL cells. In line with the MUC1-dependent regulation of ABCB1 expression, PTX substantially induced both ABCB1 mRNA (Supplementary Figure S3C) and protein (Figure 3a) in HeLa229/shCTL cells and this effect of PTX was abolished in HeLa229/shMUC1 cells. To exclude the possibility of off-target effect, we performed a rescue experiment by re-expressing MUC1-HA back to HeLa229/shMUC1-B cells. Data showed that re-expression of MUC1 restored ABCB1 expression (Figure 3b). In agreement with this notion, both mRNA (Figure 3c left) and protein levels (Figure 3c right) of ABCB1 were upregulated in HeLa229/TR cells when compared with HeLa229 parental cells. This MUC1-dependent ABCB1 regulation was further supported by that silencing MUC1 in HeLa229/TR cells resulted in reduction of both ABCB1 mRNA (Figure 3e left) and protein levels (Figure 3e right). Similar results were seen in NCI-H292 parental and NCI-H292/TR cells (Figures 3d and f). Collectively, these results indicated that MUC1 was responsible for ABCB1 induction by PTX.

### Inhibition of ABCB1 attenuates chemoresistance

**Figure 4:**
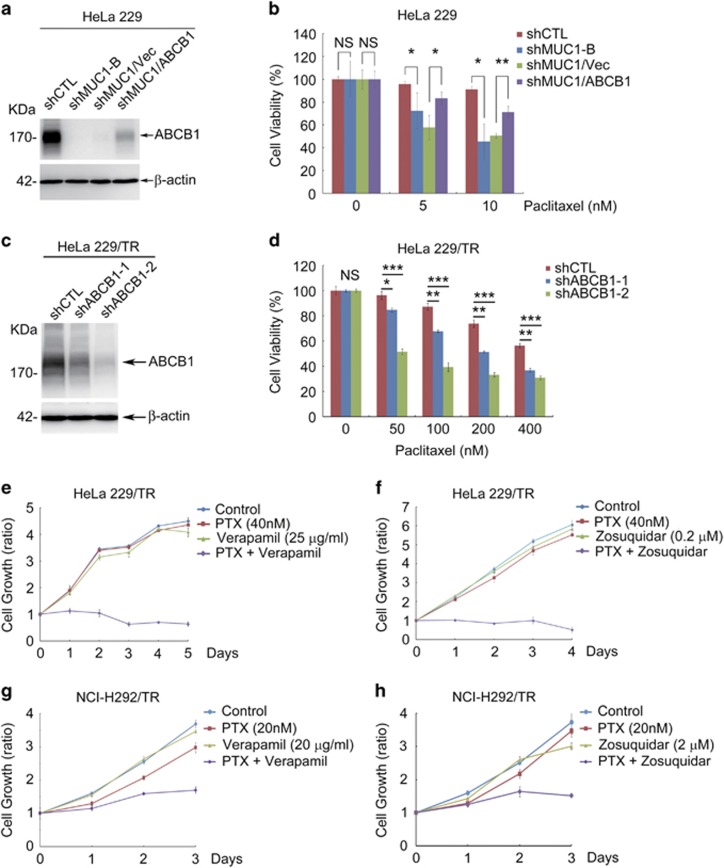
MUC1 mediates chemoresistance via ABCB1. (**a** and **b**) HeLa229/shMUC1-B cells were transiently transfected with ABCB1 plasmids (ABCB1) or vector plasmids (vec), then treated with PTX for 48 h. (**a**) Western blot was used to identify the expression of ABCB1. (**b**) CCK8 assay was carried out to test the cell viability of the cells. (**c**) Western blot was used to identify the expression of ABCB1 in HeLa229/TR/shCTL and HeLa229/TR/shABCB1 cell lines with *β*-actin as loading control. (**d**) CCK8 was carried out to identify the cell viability of HeLa229/TR/shABCB1 and its control cell line treated with PTX for 48 h. (**e** and **f**) CCK8 assays were applied to analyze the proliferation of HeLa229/TR cells treated with PTX (40 nM) combined with verapamil (25 *μ*g/ml) (**e**) or zosuquidar (0.2 *μ*M) (**f**) for indicated days. (**g** and **h**) CCK8 assays were applied to analyze the proliferation of NCI-H292/TR cells in the treatment of PTX (20 nM) in combination with verapamil (20 *μ*g/ml) (**g**) or zosuquidar (2 *μ*M) (**h**) for indicated days. Data are shown of three independent experiments, mean±S.D. (*n*=3)

To determine whether ABCB1 was responsible for MUC1-dependent resistance to PTX, we restored the expression of ABCB1 in MUC1-deficient cells. Expression of ABCB1 was indeed associated with a significant increase in resistance to PTX (Figures 4a and b). Conversely silencing ABCB1 in HeLa229/TR cells resulted in a marked increase in sensitivity to PTX (Figures 4c and d). To corroborate the results obtained with the genetic approach, we used a pharmacological method by using two specific ABCB1 inhibitors verapamil and zosuquidar. Treatment of HeLa229 with the ABCB1 inhibitors also diminished the resistance to PTX (Supplementary Figures S4A and S4B). In agreement with these observations, ABCB1 inhibitors conferred sensitivity of both HeLa229/TR (Figures 4e and f) and NCI-H292/TR (Figures 4g and h) cells to PTX. Interestingly, silencing MUC1 expression also sensitized cells to other substrates of ABCB1, including doxorubicin, vincristine, etoposide and epirubicin (Supplementary Figures S4C-S4F). These results collectively showed that ABCB1 was responsible for MUC1-induced chemoresistance in parental and drug resistance cells.

### MUC1 enhances nuclear translocation of EGFR and elevates ABCB1

**Figure 5:**
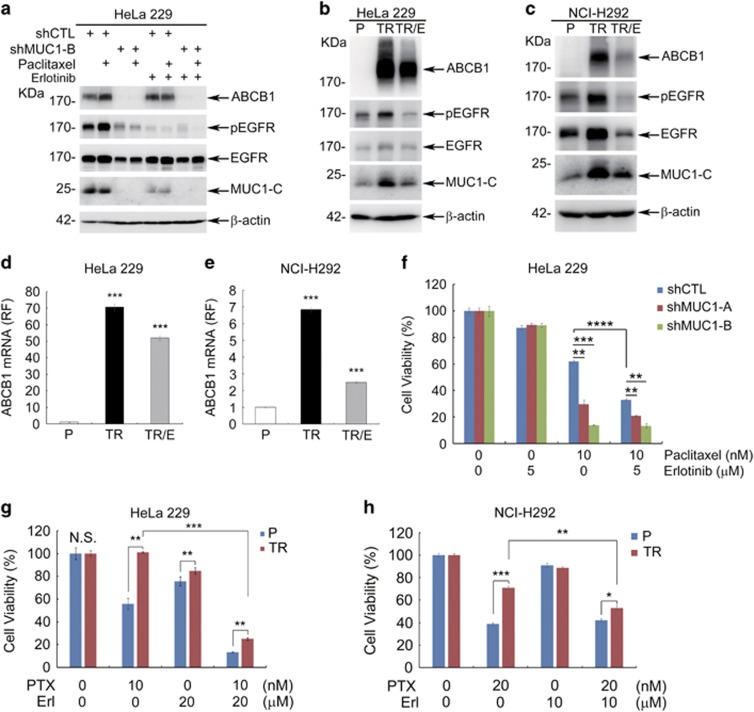
Inhibition of EGFR blocks the induction of ABCB1 and the drug resistance**.** (**a**) HeLa229/shCTL and HeLa229/shMUC1 cells were treated with PTX (10 nM) in absence or presence of EGFR inhibitor erlotinib (5 *μ*M) for 72 h. Western blot was performed to detect the expression of indicated proteins with *β*-actin as loading control. (**b**) HeLa229/TR cells were cultured in medium with 25 nM PTX for 24 h and then treated with erlotinib (20 *μ*M) (TR/E) for 48 h. Western blot was performed to detect expression of indicated proteins. (**c**) NCI-H292/TR cells were cultured in medium with 10 nM PTX for 24 h and then treated with erlotinib (10 *μ*M) (TR/E) for 48 h. Western blot was carried out to detect the indicated proteins. (**d** and **e**) RT-qPCR was carried out to identify the mRNA level of ABCB1 in HeLa229 (**d**) and NCI-H292 (**e**) cells. (**f**) The effect of combination of erlotinib (5 *μ*M) and PTX (10 nM) in HeLa229/shCTL and HeLa229/shMUC1 cells was analyzed at 48 h by CCK8 assay. (**g**) HeLa229 (P) and HeLa229/TR (TR) cells were treated with PTX (10 nM) in combination with erlotinib (Erl, 20 *μ*M); (**h**) NCI-H292(P) and NCI-H292/TR (TR) cells were treated with PTX (20 nM) in combination with erlotinib (Erl, 10 *μ*M). CCK8 assay was utilized to analyze the cell viability at 48 h. Data are shown of three independent experiments, mean±S.D. (*n*=3)

We next investigated how MUC1 upregulated ABCB1. Given the association of MUC1 with the EGFR pathway^35, 36^ and the latter in regulation of ABCB1 expression,^37, 38^ we asked whether MUC1-induced ABCB1 expression was mediated by the EGFR pathway. We tested this hypothesis by treating cells with PTX in combination with erlotinib, an inhibitor of EGFR. Erlotinib effectively blocked EGFR phosphorylation and PTX-induced ABCB1 expression in HeLa229/shCTL cells (Figure 5a). In line with these results, blocking EGFR phosphorylation with erlotinib in both HeLa229/TR cells and NCI-H292/TR cells was associated with a marked reduction of the abundance of MUC1 as well as ABCB1 (Figures 5b and c). Quantification of the transcript level revealed that erlotinib impeded PTX-induced increase of ABCB1 mRNA in HeLa229 cells (Supplementary Figure S5A) and HeLa229/TR cells (Figure 5d), as well as NCI-H292/TR cells (Figure 5e). In addition, silencing of EGFR in HeLa229/TR cells also diminished ABCB1 (Supplementary Figure S5B) and chemoresistance (Supplementary Figure S5C). These results suggested that although EGFR alone had little effect, it modulated MUC1-induced ABCB1 expression. In support of the importance of EGFR/MUC1 in transcriptional regulation of ABCB1, erlotinib significantly sensitized HeLa229 (Figure 5f) and HeLa229/TR (Figure 5g) to PTX-induced growth inhibition, as well as NCI-H292/TR cells (Figure 5h). Altogether, the results showed that MUC1/ EGFR was involved in PTX-induced ABCB1 enrichment.

**Figure 6:**
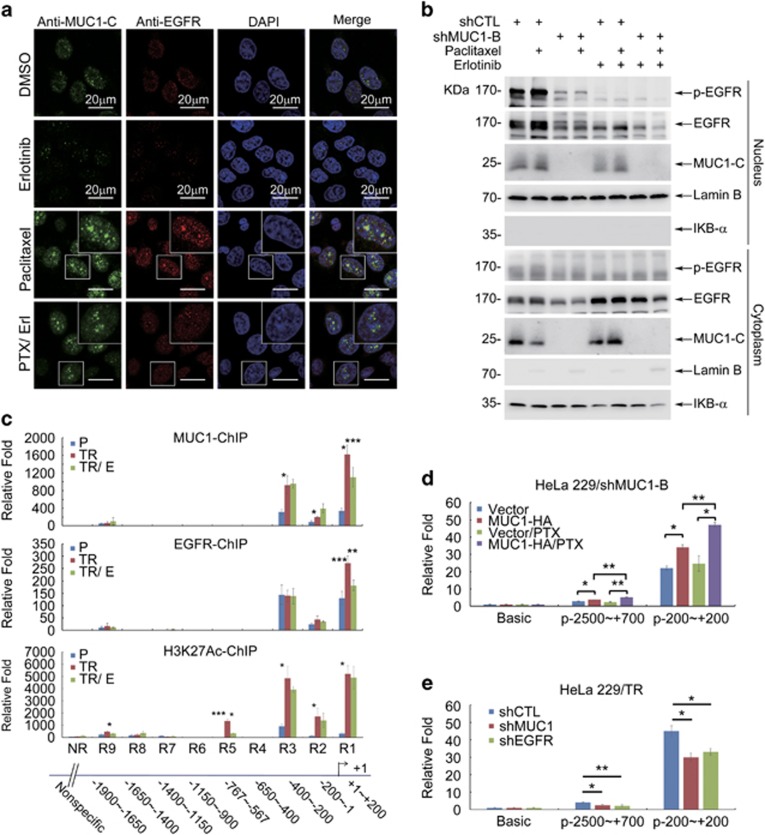
MUC1 enhances nuclear translocation of EGFR and elevates ABCB1 upon PTX treatment. (**a**) HeLa229 cells were treated with PTX (10 nM) in absence or presence of erlotinib (Erl, 5 *μ*M) for 12 h. The MUC1-C (green) and EGFR (red) were detected by immunofluorescence. Nuclei were stained with DAPI (blue). (**b**) HeLa229/shCTL and HeLa229/shMUC1 cells were treated with 10 nM PTX in absence or presence of erlotinib (5 *μ*M) for 12 h, then nuclear and cytoplasmic proteins were purified. Western blot was carried out to detect the expression of indicated proteins, Lamin B and I*κ*B-*α* were used as loading control for nuclear and cytoplasmic protein separately. (**c**) HeLa229 (P) and HeLa229/TR cells were treated with (TR/E) or without (TR) erlotinib (20 *μ*M) for 48 h, then ChIP assay was performed with anti-MUC1-C, EGFR and histone H3 (acetyl K27, H3K27Ac) antibodies, respectively. RT-qPCR was carried out to detect the potential binding sequence in the promoter of ABCB1 gene. H3K27Ac-ChIP was used as the positive control. Student’s *t*-test was performed between P and TR or TR and TR/E groups, *P<0.05, **P<0.01 and ***P<0.001. (**d**) HeLa229/shMUC1-B cells were transfected with pGL3-ABCB1 promoter or pGL3-basic plasmids (500 ng) with shMUC1-B-resistant pIRESpuro2-MUC1-HA (MUC1-HA) plasmid or vector plasmids (400 ng), then 24 h after transfection, the cells were treated with PTX (10 nM) for 12 h. Luciferase activity was detected. (**e**) HeLa229/TR/shCTL or HeLa229/TR/shMUC1 or HeLa229/TR/shEGFR cells were transfected with pGL3-ABCB1 promoter or pGL3-basic plasmids (500 ng), luciferase activity was measured at 36 h after transfection. The relative folds of luciferase activity were calculated against that with pGL3-basic plasmids. Data are shown of three independent experiments, mean±S.D. (*n*=3)

To understand how EGFR could affect MUC1-mediated ABCB1 regulation, we examined whether EGFR may interact with MUC1 by first performing a cell fractionation experiment. The result revealed that treatment with PTX was associated with an increase of both MUC1 and EGFR in the nucleus but with less change in the cytoplasm (Supplementary Figure S5D). We conducted immunofluorescence staining to confirm the result from cell fractionation experiments. Indeed, PTX treatment not only induced increased nuclear distribution but also colocalization of MUC1 and EGFR (Figure 6a). Of note, PTX-induced nuclear colocalization of MUC1 and EGFR was efficiently blocked by inhibition of EGFR (Figure 6a). The inhibitory effect of erlotinib on PTX-induced MUC1 and EGFR nuclear distribution was further confirmed in cell fractionation assay (Figure 6b). Immunoprecipitation revealed that binding of MUC1 and phosphorylated EGFR was considerably increased by PTX treatment and effectively blocked by erlotinib (Supplementary Figure S5E). Given that multiple lines of studies have implicated MUC1^26, 28, 39^ and EGFR^40^ in transcription regulation as coactivators, we next investigated whether PTX-induced interaction between MUC1 and EGFR in the nucleus could contribute to transcriptional of ABCB1. To this end, ChIP assay was carried out in both HeLa229 and HeLa229/TR cells. The result showed that MUC1 mainly bound to three regions of the ABCB1 promoter: −400 to +200, −1150 to −1400 and −1650 to −1900, which are within the same sequences as the EGFR-binding sites (Supplementary Figures S5F and S5G). H3K27Ac-binging site indicates a transcriptional activating region. Consistence with the previous results, treatment with erlotinib significantly reduced the binding of MUC1 and EGFR onto the ABCB1 promoter, in particular within the region of +1 to +200 (Figure 6c). To confirm the transcriptional regulation of ABCB1 by MUC1/EGFR, we performed luciferase assay by transfecting HeLa229/shMUC1-B cells with MUC1-HA plasmid together with ABCB1 promoter-driven luciferase reporters. MUC1 expression stimulated luciferase activity, which was further enhanced by PTX treatment. Consistence with the finding in ChIP experiment, this effect was mediated specifically in the region of −200 to +200bp (Figure 6d). In addition, silence of MUC1 or EGFR attenuated the ABCB1 transcription further revealing an important role of MUC1/EGFR in ABCB1 regulation (Figure 6e). These data collectively suggested that MUC1 activated EGFR and induced transcription of ABCB1 in HeLa229 and NCI-H292 cells.

### Co-administration of MUC1–EGFR–ABCB1 axis and PTX prevents tumor relapse

**Figure 7:**
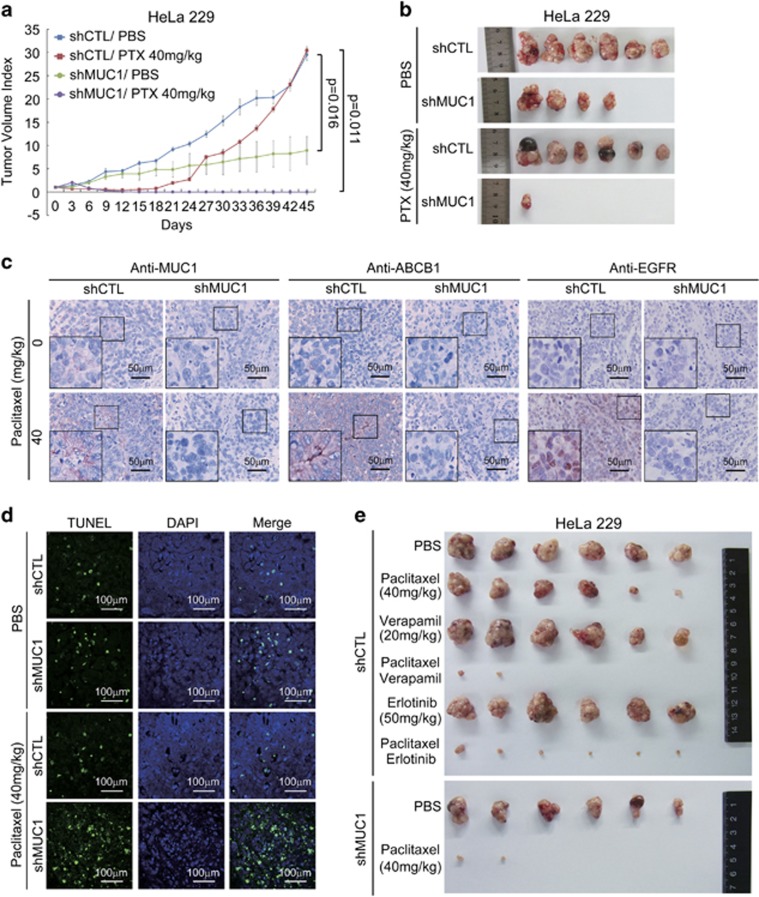
Co-administration of the inhibitors of MUC1–EGFR–ABCB1 axis and PTX prevents tumor relapse. (**a** and **b**) Six-week-old female BALB/c nude mice were subcutaneously injected with 2.5 × 10^6^ HeLa229/shCTL cells or HeLa229/shMUC1 cells in ventral flanks. When tumor reached approximately 4 mm × 4 mm, the mice were injected intraperitoneally with PTX at 40 mg/kg every three days for 15 days. The tumor sizes were measured every 3 days following PTX treatment. The tumor volume was calculated according to the formula: V=length × width^2^/2. The data indicated mean with S.E.M. of six mice in each group. (**b**) At the 45th day, all mice were killed and tumors were excised and photographed. (**c** and **d**) IHC stainings (**c**) or TUNEL assay (**d**) of tumor tissue sections were carried out. (**e**) 2.5 × 10^6^ HeLa229/shCTL cells or HeLa229/shMUC1 cells were subcutaneously injected in ventral flanks of 6-week-old female BALB/c nude mice. When the tumor reached 4 mm × 4 mm, the mice were blindly allocated into six groups and injected with PTX (40 mg/kg) in combination with verapamil (20 mg/kg) or erlotinib (50 mg/kg) intraperitoneally every 3 days for 15 days. At the 36th day, all mice were killed and tumors were excised and photographed

To investigate the contribution of MUC1 to chemoresistance *in vivo*, we generated xenograft mouse models. HeLa229/shCTL derived tumors were initially sensitive to PTX treatment, as reflected by ceased growth. However, the tumors resumed growth at day 21 after conclusion of PTX treatment (Figure 7a), consistent with acquired PTX resistance. A critical role of MUC1 in this chemoresistance was evidenced by the finding that MUC1 depletion was not only associated with reduction of tumor growth, but also with a complete prevention of tumor relapse after ending PTX treatment (Figures 7a and b). In agreement with our *in vitro* data, PTX treatment induced elevated expression levels of MUC1, ABCB1, and marked increase of EGFR nuclear localization in tumor tissues (Figure 7c). Of note were that these effects were only evident in HeLa229/shCTL tumor but not in HeLa229/shMUC1 tumor (Figure 7c), supporting a MUC1 dependency. TUNEL staining revealed that PTX treatment induced more apoptosis in HeLa229/shMUC1 tumors than that in HeLa229/shCTL tumors (Figure 7d). To examine the contribution of the MUC1/EGFR–ABCB1 axis to tumor chemoresistance, we treated the HeLa229/shCTL tumor-bearing mice with PTX in combination with verapamil or erlotinib. Similar to the sensitizing effect of shMUC1, verapamil or erlotinib substantially augmented PTX-induced inhibition of tumor growth (Figure 7e,Supplementary Figures S6A and S6B). Of note was that there was little difference in body weights of mice within groups of drug alone and combination treatment (Supplementary Figure S6C), indicating that the treatments did not cause significant toxicity. These data collectively support a critical role of the MUC1/EGFR–ABCB1 axis in acquired chemoresistance of HeLa229 cells, and moreover that targeting this axis can effectively overcome the chemoresistance.

## Discussion

Cervical cancer is the second most common malignancy that affects women worldwide with high mortality.^41^ PMC is a rare histologic type of lung malignancies.^42^ PTX is an important drug for first-line treatment of both cancers. Functional characterization of MUC1 in these two cancers demonstrates that MUC1 mediates the development of acquired chemoresistance of cancer cells. Treatment of cancer cells with chemotherapy drugs induces the expression of MUC1, which stimulates the activation and nuclear distribution of EGFR. Together, EGFR and MUC1 transcriptionally upregulate ABCB1 contributing to acquirement of chemoresistance. In support of a critical role for the MUC1/EGFR–ABCB1 axis in chemoresistance, targeted inhibition of EGFR by shRNA and erlotinib sensitizes cancer cells to chemotherapy *in vitro* and *in vivo*. Our work not only uncovers novel insight into acquired chemoresistance in cervical cancer and PMC, but also carries important therapeutic implication.

The overexpression of MUC1 in cancers and its association with poor prognosis in cancer patients led us to investigate a potential role of this oncoprotein in cancer progression.^19, 20^ Using a combination of loss- and gain-of function approach, we provided both *in vitro* and *in vivo* evidence directly linking MUC1 to acquired chemoresistance. MUC1 was induced in cancer cells upon treatment with chemotherapeutic drugs. Of particular interest was the finding that the expression of MUC1 was considerably upregulated in cancer cells that had acquired chemoresistance following long-term PTX treatment. In line with our observations, MUC1 was previously reported to associate with therapy resistance in several other cancer types, such as breast cancer^31^ and pancreatic cancer.^32^ Of note is that the MUC1 expression was relatively low in untreated or naïve tumors but significantly induced by PTX, pro-longed treatment in particular. Our results indicate that PTX treatment upregulates MUC1 transcriptionally as well as post-translationally. MUC1 promoter could be regulated by epigenetic mechanism,^43^ or by cis-acting elements, such as Sp1, AP-1-4, NF-1 and NF-*κ*B.^44^ Proinflammatory cytokines were also reported to elevate MUC1.^45^ PTX could increase transcription of MUC1 by active NF-*κ*B^46^ or proinflammatory cytokines.^47^ Contrast with transcriptional regulation of MUC1, much less is known about post-translationally regulation of MUC1. Further studies will be necessary to investigate how chemotherapeutic drugs post-translationally upregulate MUC1 expression.

MUC1 has been implicated in modulating cellular sensitivity to therapy. It was previously reported that MUC1 conferred pancreatic cancer cells chemoresistance by upregulating MRP1.^32^ We identified ABCB1 as an important factor mediating MUC1-dependent chemoresistance in cervical cancer and PMC. ABC family proteins are often involved in multidrug resistance in cancer.^34^ Among the nine PTX-related ABCs, we found ABCB1 protein being the only one that was selectively induced by MUC1, although mRNA levels of ABCC1 and ABCC5 were also elevated by MUC1. Indeed, inhibition of ABCB1 with complementary pharmacological and genetic approaches substantially diminished chemoresistance of cancer cells. The results not only uncover a novel mechanism of MUC1-induced chemoresistance but also implicate ABCB1 as a potential therapeutic target in cancer cells.

To elucidate the mechanism how MUC1 regulates ABCB1, we directed our attention to the EGFR pathway because the latter is not only implicated in ABCB1 regulation^37, 38^ but also associated with MUC1.^35, 36^ Recent studies showed that EGFR was involved in chemoresistance in many cancers via regulating ABCB1 and ABCG2.^37, 38, 48, 49^ A dynamic interaction between MUC1-C and EGFR has been reported under various contexts with a general trend of mutual functional augmentation. MUC1 promotes EGFR-dependent activation of the PI3K-AKT pathway,^22, 50^ and regulates localization of EGFR to the nucleus,^36^ as well as stimulates EGFR expression by binding to the EGFR promoter. Consistent with published work, we found that increased MUC1 expression in cervical cancer cells was associated with increased phosphorylation and total level of EGFR (Figures 5a and 6b). PTX treatment stimulated activity and nuclear localization of both MUC1 and EGFR. Importantly, suppression of EGFR by inhibitor considerably repressed PTX-induced ABCB1 expression, and reversed MUC1-mediated resistance to PTX (Figure 5f). In addition, silencing of either MUC1 or EGFR in HeLa229/TR cells results withdrew ABCB1 and chemoresistance (Figure 3e,Supplementary Figures S2B, S5B and S5C). The MUC1/EGFR complex formation was further confirmed by co-IP assay (Supplementary Figure S5E). These data support a model of MUC1/EGFR–ABCB1 axis in MUC1-induced chemoresistance. Of note is that erlotinib also markedly diminished PTX-induced nuclear distribution of MUC1 and EGFR, which would impede their transcriptional activity. Indeed, ChIP assay revealed that treatment with erlotinib substantially decreased the binding of MUC1 and EGFR to the ABCB1 promoter, providing important molecular insight into MUC1-mediated chemoresistance. The most enrichment of luciferase activity was found in region of −200 to +200, which included binding sites for many important transcription factors, such as STAT3 (+64 to +72), FOXO3a (−181 to +68), p53 (−49 to −40; −72 to −62), InvMED (−106 to −100), NFR1/2 (−123 to −115), C/EBP (−147 to −135), NF-*κ*B (−167 to −158), AP-1 (−122 to −116) and *β*-catenin (−228 to +31). The identification of the binding site for MUC1/EGFR in ABCB1 promoter is instrumental for elucidation of the mechanism by which that MUC1/EGFR regulates transcriptional activation of ABCB1.^51^

We demonstrated that combined use of EGFR inhibitors with ABCB1 substrate may represent a promising strategy to reverse ABCB1-mediated chemoresistance. Several small molecular tyrosine kinase inhibitors (TKIs), such as gefitinib,^52, 53^ erlotinib^54, 55^ and lapatinib,^56^ have been approved by the FDA for the treatment of NSCLC where EGFR is often activated via amplification or mutation.^57^ Interestingly, it was reported that acquired resistance to PTX in ovarian carcinoma cells is also associated with enhanced sensitivity to HER1/EGFR inhibitors, which correlates with increased HER1/EGFR expression.^58^ In line with this finding, we found that EGFR was initially undetectable in cervical tumor but induced by PTX treatment (Figure 7c), which was accompanied with increased MUC1/ABCB1 expression and therapeutic resistance. Our data suggest a close correlation between chemoresistance and EGFR activation in cervical tumor and PMC. Consistent with this notion is our data showing that EGFR was activated by MUC1 in PTX-resistant cancer cells and more importantly, MUC1 inhibition resulted in diminished activity of EGFR and reduced expression of ABCB1 leading to reversal of PTX resistance. In our cervical cancer xenograft mouse model, co-administration of PTX with EGFR inhibitor erlotinib significantly prevented the tumor growth and relapse, which was associated with reduced expression of MUC1 and ABCB1, and diminished activity of EGFR. Our data implicate that EGFR inhibitors in addition to combination with PTX can be used to overcome acquired chemotherapy resistance in MUC1-positive cancer.

Many pathways were reported to regulate ABCB1 expression. For instance, the inhibition of p38/MAPK reduced the activator protein-1 (AP-1) activity and ABCB1.^59^ Activation of JNK/c-Jun/AP-1 reduces ABCB1 mRNA expression.^60, 61^ Our data showed that EGFR inhibitors did not completely inhibit interaction of MUC1 and ABCB1 promoter, suggesting the possible effect of MUC1 on other pathway in addition to EGFR.

Our findings also have implications in treating other cancers, as MUC1 has been shown to be upregulated in many PTX-resistant tumors including ovarian cancer and gastric cancer (Supplementary Figure S7) based on ONCOMINE database. Further studies will be needed to investigate whether our findings in this study can be translated to other cancers in which MUC1 and ABCB1 are positive.

## Materials and methods

### Cell culture

HeLa229, HEK293T and NCI-H292 were provided by Cell Bank, Type Culture Collection, Chinese Academy of Sciences (Shanghai, China) and Fu Heng Biology Company (Shanghai, China), respectively. HeLa229 and NCI-H292 cells were cultured in RPMI1640 (Corning, NY, USA) with 10% fetal bovine serum (Gibco, Grand Island, NY, USA), and HEK293T was cultured in DMEM with 10% fetal bovine serum (Gibco) supplemented with 100 U/ml penicillin and 100 *μ*g/ml streptomycin, and maintained in humidified atmosphere of 5% CO_2_ at 37 °C. All cell lines are routinely tested and free of mycoplasma.

### Generation of PTX-resistant cell lines

To generate PTX-resistant HeLa229/TR cells, HeLa229 cells were exposed gradually to 5, 10, 15, 20 and 25 nM PTX, and each treatment lasted 9 days. PTX-resistant NCI-H292/TR cells were generated by being exposed gradually to 2.5, 5, 10 nM PTX.

### Plasmids and transfection

HeLa229/shCTL or HeLa229/shMUC1-A/B cells were accomplished by transfecting HeLa229 cells with pRNAU6.1-shCTL or pRNAU6.1-shMUC1-A/B using X-treme GENE HP DNA Transfection Reagent (Roche Applied Science, Basel, Switzerland) and selected by 500 *μ*g/ml G418. Two target sequences for MUC1 are shown as following: shMUC1-A: 5′-AAGGTACCATCAATGTCCACG-3′, and shMUC1-B: 5′-AAGTTCAGTGCCCAGCTCTAC-3′. The control shRNA (shCTL) sequence is 5′-CGCTTACCGATTCAGAATGG-3′. PGIPZ-puromycin lentiviral plasmids were from the Thermo Scientific Open Biosystems GIPZ Lentiviral shRNAmir Library (Thermo Fisher Scientific, Waltham, MA, USA). The target sequences were as following: shMUC1-1: 5′-CCAGCACCGACTACTACCA-3′ shMUC1-2: 5′-GAAATGTTTTTGCAGATTT-3′ shABCB1-1: 5′-CAGATAATATTAAGGGAAA-3′ shABCB1-2: 5′-AGATGATGTCTCCAAGATT-3′ shEGFR-1: 5′- AGGAACTGGATATTCTGAA-3′ ShEGFR-2: 5′-AGATCAGAAGACTACAAAA-3′. Nonsense control sequence is 5′-CTCGCTTGGGCGAGAGTAA-3′. The shRNA plasmids and packaging plasmids (PM2G and PSPAX2) were co-transfected into HEK293T cells. The viral supernatants were collected 48 h after transfection. The HeLa229/TR and NCI-H292/TR cells were infected with shMUC1 (shMUC1-1/shMUC1-2), shABCB1 (shABCB1-1/shABCB1-2), shEGFR (shEGFR-1/shEGFR-2) and shCTL lentivirus with medium containing 1 *μ*g/ml polybrene (Santa Cruz Biotechnology, Santa Cruz, CA, USA). After 48 h, cells were selected with 3 *μ*g/ml puromycin (HeLa229/TR) or 1.5 *μ*g/ml puromycin (NCI-H292/TR). To construct MUC1-expressing plasmid, the full length of MUC1 with HA tag at C-terminal was cloned into pIRESpuro2 vector. To construct shMUC1-B-resistant MUC1-HA-expressing plasmid pIRESpuro2-MUC1-HA (MUC1-HA), the target sequence of shMUC1-B was synonymous mutated from 5′-aagttcagtgcccagctctac-3′ to 5′-aagCtcCgtgccTagctcGac-3′. The pHa-MDRwt expression plasmid (ABCB1 plasmid) was a gift from Dr. Michael Gottesman (Addgene, Cambridge, MA, USA; plasmid).

Transient transfections were performed with Lipofectamine 2000 reagent (Invitrogen, Carlsbad, CA, USA) or X-treme GENE HP DNA Transfection Reagent (Roche Applied Science, Basel, Switzerland) according to the manufacturer’s instructions.

### Cell proliferation/viability assay and IC50 values

Cell viability was determined using the cell counting kit 8 (CCK8) according to the manufacturer’s protocol (Dojindo Molecular Technologies, Kumamoto, Japan). Briefly, 6000–10000 cells per well (for drug viability assay) were seeded in 96-well plates, cultured overnight and then treated with different drugs. After relevant treatment, CCK8 was added and incubated for additional 2 h, then the absorbance at wavelength of 450 nm was measured by Synergy H4 Hybred Reader (BioTek Instruments, Winooski, VT, USA). Relative growth inhibition and the half-maximal inhibitory concentration (IC50) values were calculated by nonlinear regression analysis using the GraphPad Prism 6.0 software (GraphPad Software, Inc. La Jolla, CA, USA). Each experiment was repeated three times with triple samples.

### Drugs and antibodies

The following drugs and antibodies were used in our experiments: PTX, vincristine, doxorubicin and verapamil, cycloheximide, etoposide (Sigma-Aldrich, St. Louis, MO, USA), zosuquidar, erlotinib and AG-1478 (Selleck Chemicals, Houston, TX, USA), anti-MUC1-C antibody (Thermo Scientific, Hudson, NH, USA), anti-MUC1-N antibody,^17^ anti-HA-tag, anti-ABCB1, anti-phospho-EGFR 1068 antibodies (Cell Signaling Technology, Danvers, MA, USA), anti-ABCBG2 antibody (GeneTex, Irvine, CA, USA), anti-ABCC1 antibody (Bioworld Technology, Inc., Louis Park, MN, USA), anti-ABCC2 and anti-EGFR antibodies (Proteintech Group, Chicago, IL, USA), anti-ABCC3 and anti-ABCC5 (Cusabio Biotech, Wuhan, China), anti-Lamin B and anti-I*κ*B-α antibodies (Santa Cruz Biotechnology), anti-histone H3 (acetyl K27) antibody (Abcam, Cambridge, MA, USA), horseradish peroxidase (HRP)-linked secondary antibody (Cell Signaling Technology) and anti-*β*-actin antibody (Merck Millipore Billerica, MA, USA).

### Western blot

Cells were collected by trypsinization and lysated in NETN 150 lysis buffer (0.5% NP-40, 20 mM Tris (pH 8.0), 150 mM NaCl, 6 mM EDTA). The proteins were quantified by Bradford. Twenty microgram protein was separated by SDS-PAGE, and transferred to the nitrocellulose membrane (Axygen, Tewksbury, MA, USA). After blocking in 5% non-fat milk for 1 h at room temperature, the membrane was incubated with primary antibodies overnight at 4 °C, followed by HRP-linked secondary antibody. ImmobilonTM Western Chemiluminescent HRP Substrate kit (Millipore Corporation, Billerica, MA, USA) was used for detection.

### Quantitative real-time PCR (RT-qPCR)

Total RNA was extracted using TriPure Isolation Reagent according to the manufacturer’s protocol (Roche). Complementary DNA was synthesized using the M-MLV Reverse Transcriptase synthesis kit (Promega, Madison, WI, USA). RT-qPCR was carried out with Power SYBR Green PCR Master mix kit according to the manufacturer’s instructions (Applied Biosystems, Warrington, UK). Amplifications were performed in ABI PRISM 7500 Sequence Detection System (Applied Biosystems). Relative transcript quantities were calculated using the ΔΔCt method with *β*-actin as the endogenous reference gene. Each result was repeated three times. The primers are from PrimerBank.

Primer sequences for RT-qPCR:


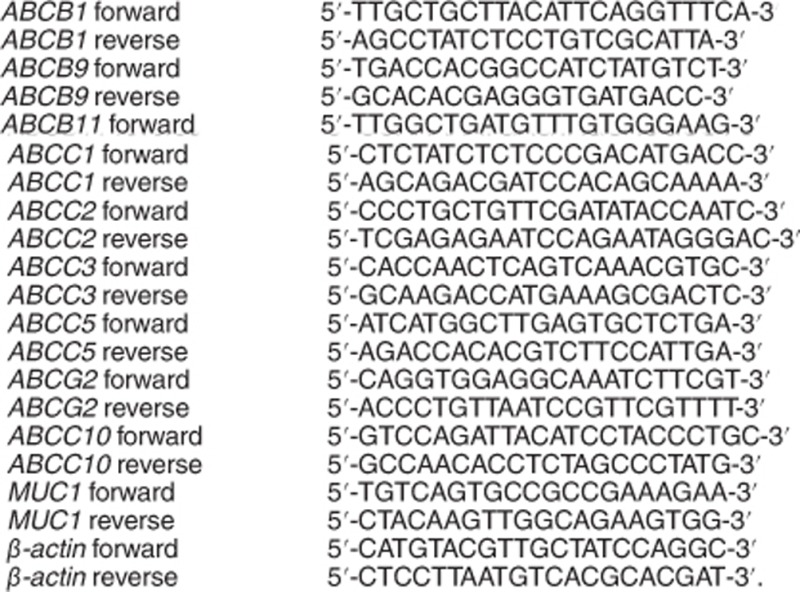


### Nuclear and cytoplasmic extraction

The nuclear and cytoplasmic proteins were purified by using NE-PER Nuclear and Cytoplasmic Extraction Reagents (Pierce Biotechnology, Inc., Rockford, IL, USA) according to the manufacturer's instructions. The nuclear and cytoplasmic proteins were then quantified by Bradford, and further analyzed by western blot.

### Immunofluorescence staining

Cultured cells were rinsed with room temperature PBS once, and fixed with cold 4% formaldehyde for 30 min, and wash with PBS for three times. Cells were permeabilized with cold methanol for 20 min and incubated with the primary antibodies overnight at 4 °C after 5% BSA blocking. The cells were incubated with fluorescent secondary antibodies (Jackson ImmunoResearch, West Grove, PA, USA) in 5% BSA for 1 h at 37 °C. Nuclei were stained with DAPI (Vector Laboratories, Burlingame, CA, USA). A confocal microscope (Nikon, Tokyo, Japan) was used to observe all stained slices.

### Chromatin immunoprecipitation

HeLa229 and HeLa229/TR cells were seeded on 150 mm cell culture dishes. Cells were washed with room temperature PBS and fixed with 1% formaldehyde in PBS for 10 min at 37 °C. Then, cells were rinsed with ice cold PBS and harvested by spin. The cell pellets were resuspended in lysis buffer (1% SDS, 5 mM EDTA, 50 mM Tris.HCl (pH 8.0) with protease inhibitor), and sonicated. Perform immunoclearing by incubating chromatin with sheared salmon sperm DNA, pre-immune serum and protein A sepharose beads for 2 h at 4 °C. The supernatant was subject to immunoprecipitation with MUC1, EGFR or histone H3 (acetyl K27) antibodies for 6 h at 4 °C, followed by addition of protein A sepharose beads, salmon sperm DNA for 1 h. Sepharose beads were harvested and washed sequentially in TSEI (0.1% SDS, 1% Triton X-100, 2 mM EDTA, 20 mM Tris.HCl (pH 8.0), 150 mM NaCl), TSEII (0.1% SDS 1% Triton X-100, 2 mM EDTA, 20 mM Tris.HCl (pH 8.0), 500 mM NaCl), buffer III (0.25 M LiCl, 1% NP-40, 1% deoxycholate, 1 mM EDTA, 10 mM Tris.HCl (pH 8.0)) and TE buffer. DNA was eluted from the beads with elution buffer (1% SDS, 0.1 M NaHCO_3_). The elution was heated at 65 °C overnight to reverse the formaldehyde cross-link, and purified with the QIAquick PCR purification kit (QIAGEN GmbH, Hilden, Germany).

Negative primers located at 167-kb upstream of ABCB1. Primers for ChIP:


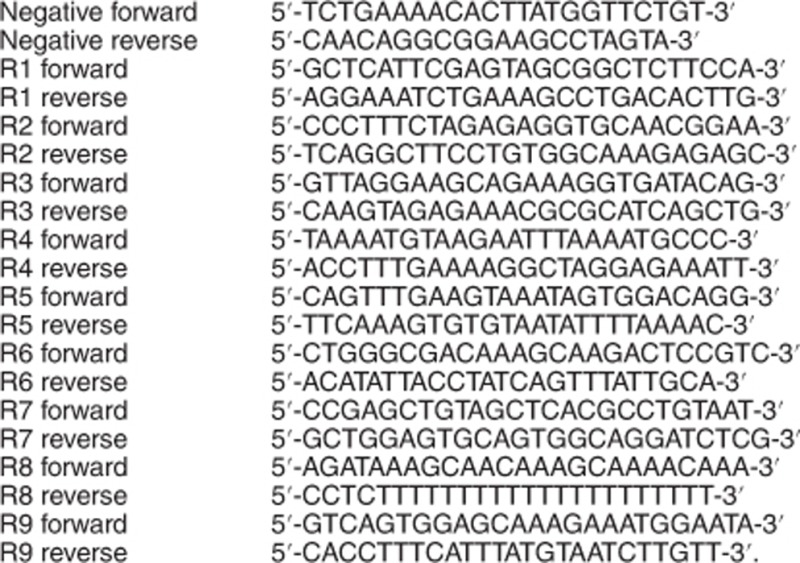


### Co-immunoprecipitation assay

HeLa229 and HeLa229/TR cells were lysed in NETN 150 lysis buffer with 1 mM PMSF and 20 mM phosphatase inhibitor cocktail tablets (Roche Diagnostics GmbH, Mannheim, Germany). Cell lysate was precleared with 30 *μ*l protein A/G plus agarose (Santa Cruz Biotechnology) at 4 °C for 2 h. The supernatant was incubated with MUC1-C-terminal antibody and hamster IgG antibody (Santa Cruz Biotechnology) overnight at 4 °C with rotation. In all, 20 *μ*l protein A/G plus agarose was added to cell lysate and incubated for 2 h at 4 °C with rotation. The immunoprecipitates were collected by centrifugation and washed with NETN 150 buffer for three times.

### Luciferase assay

HeLa229 cells were transfected with luciferase reporter plasmids pGL3-MUC1-promoter (500 ng) or pGL3-ABCB1-promoter (500 ng) together with pGL3-SV40-Renilla (10 ng). Thirty-six hours later, cells were collected and analyzed by the Dual-Luciferase Assay system (Promega) according to the manufacturer's instructions.

pGL3-MUC1-promoter luciferase reporter plasmid was generously provided by Dr. Donald Kufe at Dana-Farber Cancer Institute Harvard Medical School Boston, MA, USA.

The human ABCB1 promoter (-2500 to +700) was amplified with primers (up: 5′-GGGGTACCGCAAGGGGACCAGGTAGGTTTCATC-3′, down: 5′-CTAGCTAGCCTAGTTACCTTTTATTGT-3′), the human ABCB1 promoter (−200 to +200) was amplified with primers (up: 5′-CTAGCTAGCAGGAAATCTGAAAGCCTGAC-3′, down: 5′-GGGGTACCCCCTTTCTAGAGAGGTGCA-3′), and then cloned into pGL3-basic vector (Promega) to construct pGL3-ABCB1-promoter (−2500 to +700) luciferase reporter and pGL3-ABCB1-promoter (−200 to +200) luciferase reporter.

### TUNEL assay

To detect apoptotic cells in tumor tissue, the samples were fixed in 4% paraformaldehyde immediately, and tumor tissue sections were stained by the TUNEL kit (Promega) according to the manufacturer's instructions. Fluorescence signals were captured on a Confocal microscope (Nikon).

### Histological and immunohistochemical staining (IHC)

Tumor tissues were fixed in 4% paraformaldehyde and then dehydrated before embedding in paraffin. In all, 6 *μ*m tissue were sliced and stained according to the standard procedure with eosin and hematoxylin. Paraffin-embedded tumors were de-paraffinized with xylene for 5 min twice, and then treated with gradient ethanol for 3 min for each time. The tissue sections were then heated to 92–98 °C in 10 mM citrate buffer (pH 6.0) for 35 min for antigen retrieval. To increase tissue permeability, tissue sections were treated in PBST (0.2% Triton X-100) for 10 min. Tissue sections were treated with 3% hydrogen peroxidase in methyl alcohol for 10 min and then incubated with blocking serum for 1 h at room temperature. Tissue sections were incubated with primary antibody at 4 °C overnight. IHC staining was performed with ABCB1 and MUC1-C, EGFR antibodies. The staining procedure followed the manufacturer’s instructions for the ABC staining system (Santa Cruz Biotechnology).

### Animal experiments

All animals were handled according to the Guide for the Care and Use of Laboratory Animals’ and the Principles for the Utilization and Care of Vertebrate Animals’. All animal experiments consulted the ARRIVE guidelines for animal.^62^ Research was approved by the Institutional Animal Care and Use Committee at Shanghai Jiaotong University School of Medicine. Investigators were blinded to the group allocation.

Tumor cells were injected subcutaneously in ventral flanks of 6-week-old female BALB/c nude mice. When the tumors reached approximately 4 mm × 4 mm, tumor-bearing mice were randomly assigned (*n*=6 per group) and treated with different drugs.

Mice injected with 2.5 × 10^6^ of HeLa229/shCTL or HeLa229/shMUC1 cells were assigned blindly into two groups for drug resistance experiments with six mice for each group: mice in group 1 were treated with PBS as control, and mice in group 2 were treated with PTX (40 mg/kg). PTX (Taxol; Bristol-Myers Squibb, Princeton, NJ, USA) was appropriately diluted in PBS before treatments. Drugs were injected intraperitoneally every 3 days. The total treatment period was 15 days, 30 days after the final administration, the mice were killed.

Mice injected with 2.5 × 10^6^ HeLa229/shCTL were blindly assigned into six groups for combination drug resistance experiments with six mice for each group: groups 1 and group 2 were treated as above, group 3 were treated with verapamil (20 mg/kg), group 4 were treated with verapamil and PTX, group 5 were treated with erlotinib (50 mg/kg) and group 6 were treated with erlotinib and PTX. Mice injected with 2.5 × 10^6^ HeLa229/shMUC1 were assigned into two groups as above with six mice for each group. Erlotinib and verapamil (Selleck Chemicals) were prepared according to the manufacturer's instructions, then diluted in PBS. Drugs were injected intraperitoneally every 3 days. Tumor growth was monitored by caliper rule every 3 days, and the total treatment period was 15 days for combination drug treatment. Three weeks after the final administration, the mice were killed. The volume was calculated according to the formula: V=length × width^2^/2.

### Statistical analysis

All statistical analyses were performed by GraphPad Prism 6 (GraphPad Software, La Jolla, CA, USA). Each result is represented as the mean±S.D. of three independent experiments(*n*=3) and two-sided student’s *t*-test was performed to evaluate the differences between linked groups, *P-*values <0.05 were considered as statistically significant. *P<0.05, **P<0.01, ***P<0.001 and ****P<0.0001.
