# Supplementary material for: MUC1 induces acquired chemoresistance by upregulating ABCB1 in EGFR-dependent manner
Source: Cell Death Dis. 2017 Aug 10;8(8):e2980–. doi: 10.1038/cddis.2017.378 (PMC5596566; doi:10.1038/cddis.2017.378)
Supplement: Supplementary Figures [file cddis2017378x1.docx]

**Supplemental information**

**Figure legend for supplementary figures**

**Figure S1.** (A-B) The mRNA levels of MUC1 of indicated types of cervical (A) or lung (B) cancer patients’ sample from Scotto Cervix 2 or Garber Lung ONCOMINE microarray databank respectively. Y-axis shows the log2 median-centered intensity of MUC1. (C-D) HeLa229 parental cells (P) and paclitaxel-resistant HeLa229 (TR) cells (C) or NCI-H292 parental cells (P) and NCI-H292 paclitaxel resistance (TR) cells (D) were treated with different concentrations of paclitaxel for 48 hours and cell viability was measured by CCK8 assay. IC50s were calculated by GraphPad Prism 6.0 software. (E) HeLa229 cells were treated with 5 nM paclitaxel (PTX) and harvested at indicated time point. HeLa229 cells without paclitaxel treatment were also collected at day 4 as the control (CTL). Western blot was carried out to identify the accumulation of MUC1 protein. (F) HeLa229 cells were treated with DMSO or 10 nM paclitaxel for 48 hours, then expose to cycloheximide (CHX)(50 μg/ml) for indicated time. Western blot was carried out to identify the expression of MUC1.

**Figure S2.** (A)IC50s of HeLa229/TR/shCTL and HeLa229/TR/shMUC1 cells treated with paclitaxel (PTX) for 48 hours were identified. (B)IC50s of NCI-H292/TR/shCTL and NCI-H292/TR/shMUC1 cells treated with paclitaxel (PTX) for 48 hours were identified. (C) IC50s of HeLa229/shCTL and HeLa229/shMUC1 cells treated with paclitaxel (PTX) for 48 hours were identified. (D) CCK8 assay was carried out to detect the proliferation of HeLa229/TR/shCTL and HeLa229/TR/shMUC1 cells. (E) CCK8 assay was carried out to detect the proliferation of HeLa229/shCTL and HeLa229/shMUC1 cells.

**Figure S3.** (A-B) The mRNA and protein levels of ATP binding cassette (ABC) transporters genes were identified by RT-qPCR (A) and western blot (B) in HeLa229/shCTL and HeLa229/shMUC1 cells. (C) HeLa229/shCTL and HeLa229/shMUC1 cells were treated with 0, 5, 10 nM paclitaxel for 48 hours, RT-qPCR was used to detect the mRNA of ABCB1 with β-actin as internal control. * was compared to 0 nM paclitaxel treated group among same cells; # was compared to shCTL group between different cells. */# P<0.05, **/## P<0.01, and ### P<0.001.

**Figure S4.** (A-B) CCK8 assays were used to analyze the cell viability of HeLa229/shCTL or HeLa229/shMUC1 cells which were treated with paclitaxel (PTX, 10 nM) in combination with ABCB1 inhibitor verapamil (VPM, A) or zosuquidar (Zos, B) for 48 hours. (C-F) CCK8 assays were applied to detect cell growth inhibition of HeLa229/shCTL and HeLa229/shMUC1 cells with the treatment of doxorubicin(C), vincristine(D), etoposide(E), epirubicin(F) for 48 hours. IC50s of indicated drugs were calculated by GraphPad Prism 6.0 software.

**Figure S5.** (A) HeLa229 cells were treated with 5 nM paclitaxel in absence or presence of EGFR inhibitor erlotinib (5 μM) or AG1478 (5 μM) for 48 hours. RT-qPCR was carried out to test the mRNA level of ABCB1. (B) HeLa229/TR cells stably expressing PGIPZ-shEGFR (shEGFR) or PGIPZ-shCTL (shCTL) lentivirus were selected, and western blot was utilized to identify the expression of ABCB1 and EGFR with β-actin as loading control. (C) CCK8 assay was carried out to identify the cell viability of HeLa229/TR/shEGFR and its control cell line treated with paclitaxel for 48 hours.(D) HeLa229 cells were treated with 10 nM paclitaxel(PTX) for indicated times, and the nuclear (N) and cytoplasmic (C) proteins of these cells were purified as described in Materials and Methods. Western blot was carried out to detect the expression of indicated proteins, Lamin B and IκB-α were used as loading control for nuclear and cytoplasmic protein separately. (E) MUC1 was immunoprecipitated (IP) by MUC1-C terminal antibody in HeLa229 parent (P) and HeLa229/TR cells treated with (TR/E) or without (TR) erlotinib (20 μM) for 48 hours, then western blot was carried out to detect MUC1-C and EGFR protein level. (F-G) ChIP assays were performed with MUC1-C terminal, EGFR and Histone H3 (acetylated at K27, H3K27Ac) antibodies in HeLa229 (E) and HeLa229/TR (F) cells. PCR was carried out to detect the potential promoter sequence these proteins binding. H3K27Ac-ChIP was used as the positive control.

**Figure S6.** (A) Six-week-old female BALB/c nude mice were subcutaneously injected with 2.5 x 10^6^ HeLa229/shCTL cells or HeLa229/shMUC1 cells in ventral flanks. When tumor reached approximately 4 mm x 4 mm, the mice were injected intraperitoneally with indicated drugs for 15 days. The tumor sizes were measured every 3 days. The tumor volume was calculated according to the formula: V=length*width^2^ /2. The data indicated means with S.E.M. of six mice in each group. (B-C) At day 36, mice were euthanized and tumors were excised. Tumor weight (B) and mice weight (C) were measured. Data are shown as mean ± S.E.M of 6 mice. Lines indicate mean and S.E.M. The p values between line-linked groups were calculated by student t-test.

**Figure S7.** (A)The mRNA levels of MUC1 in ovarian cancer cell lines were analyzed. The intermediate and resistant ovarian cancer cell lines have higher levels of MUC1 than the sensitive ones. (B)The mRNA levels of MUC1 in gastric cell lines were analyzed. MUC1 has higher expression in paclitaxel intermediate sensitive and resistant gastric cancer cell lines than the sensitive cell lines. These microarray data from ONCOMINE databank include 917 human cell line samples. Y-axis shows the log2 median-centered intensity of MUC1.

**Supplementary figures**

**
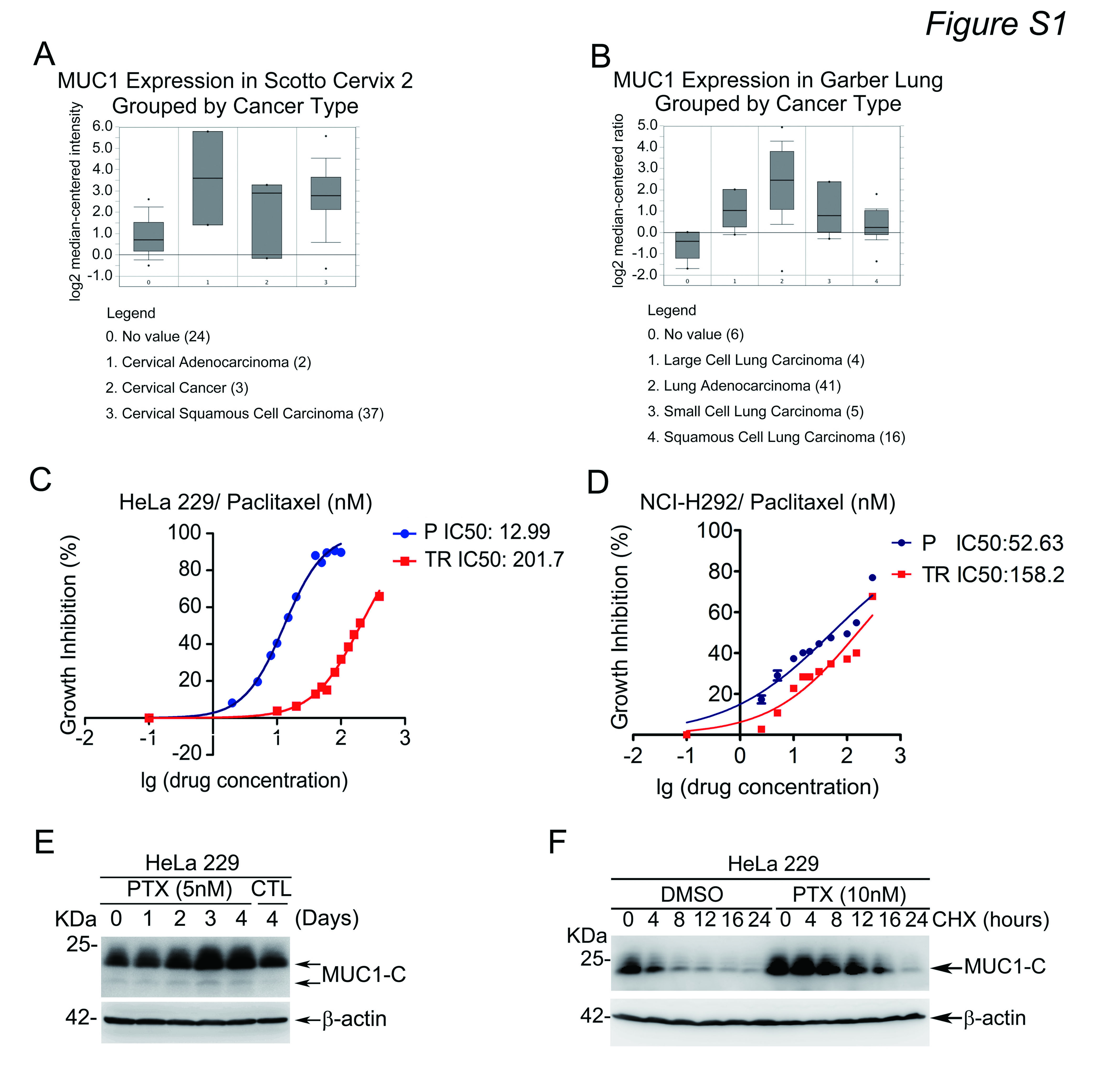
**

**
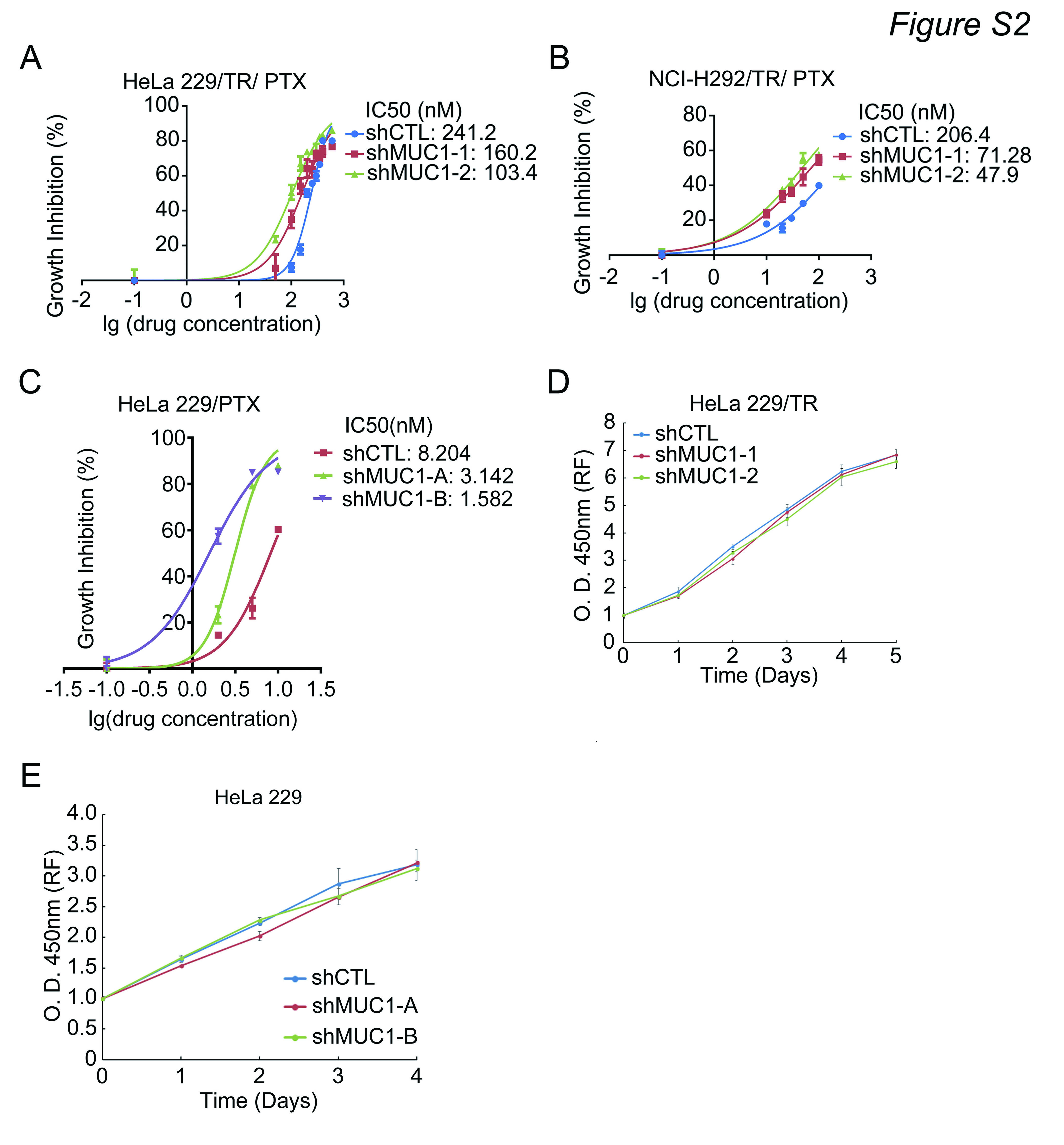
**

**
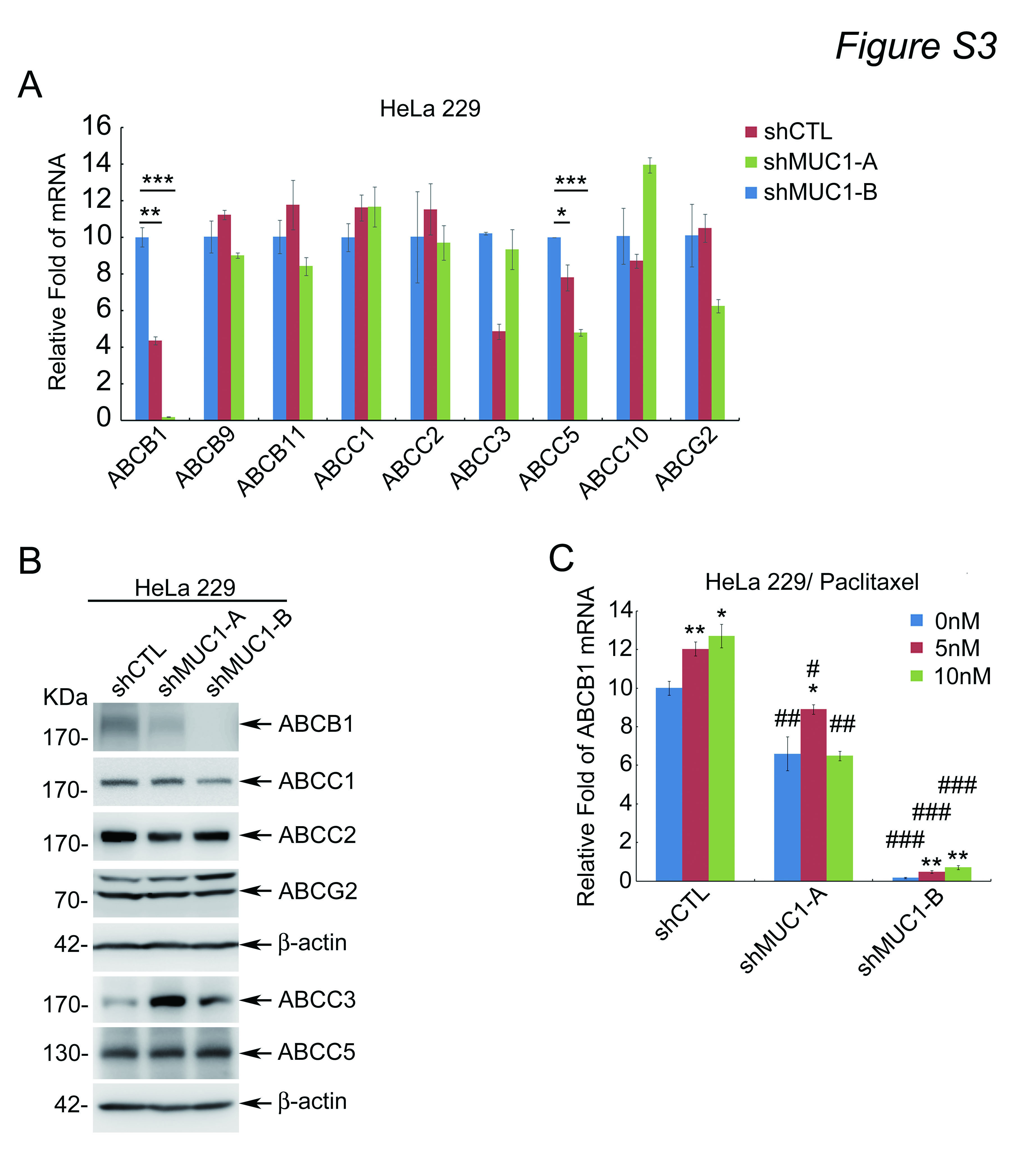
**

**
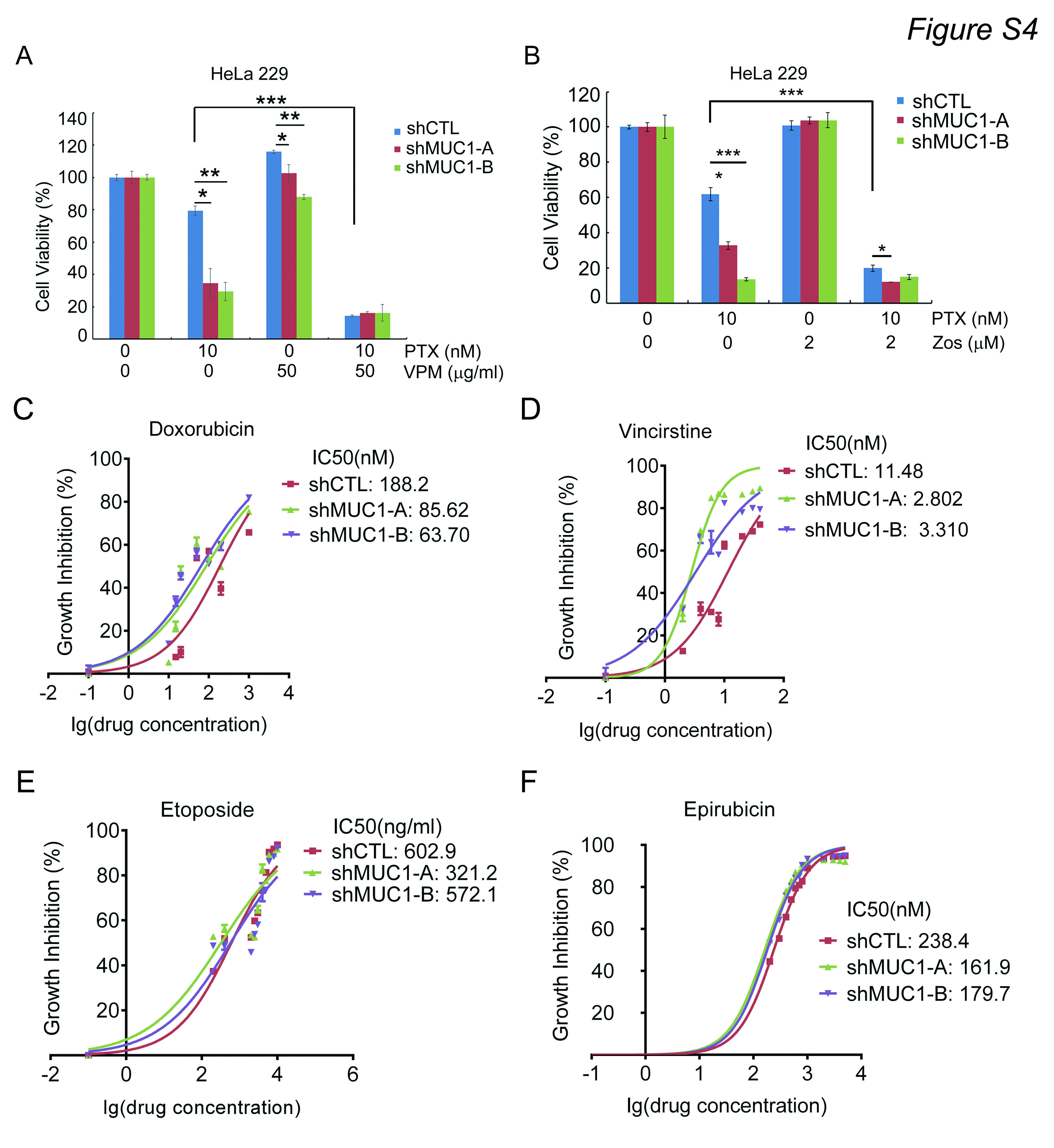
**

**
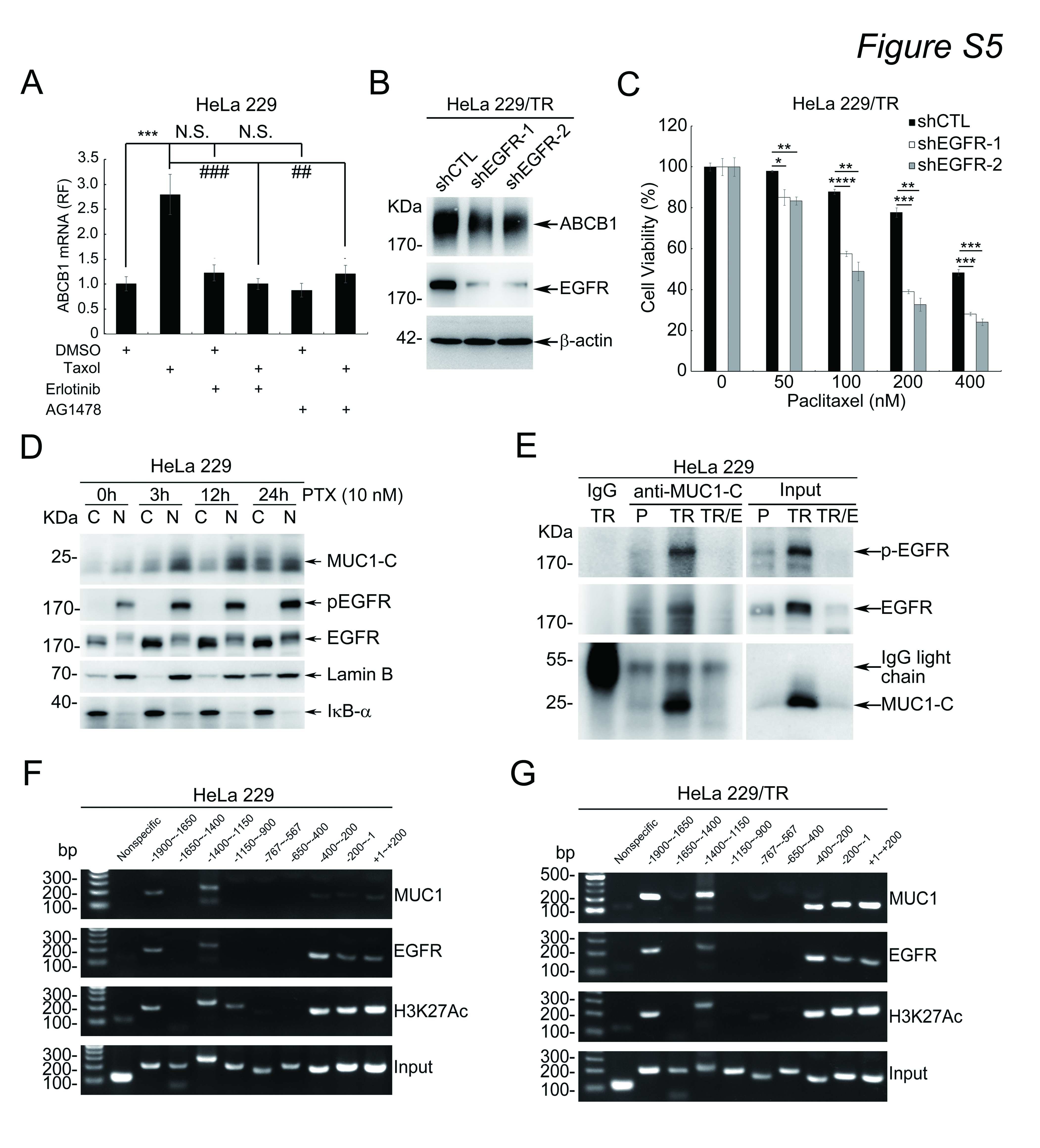
**

**
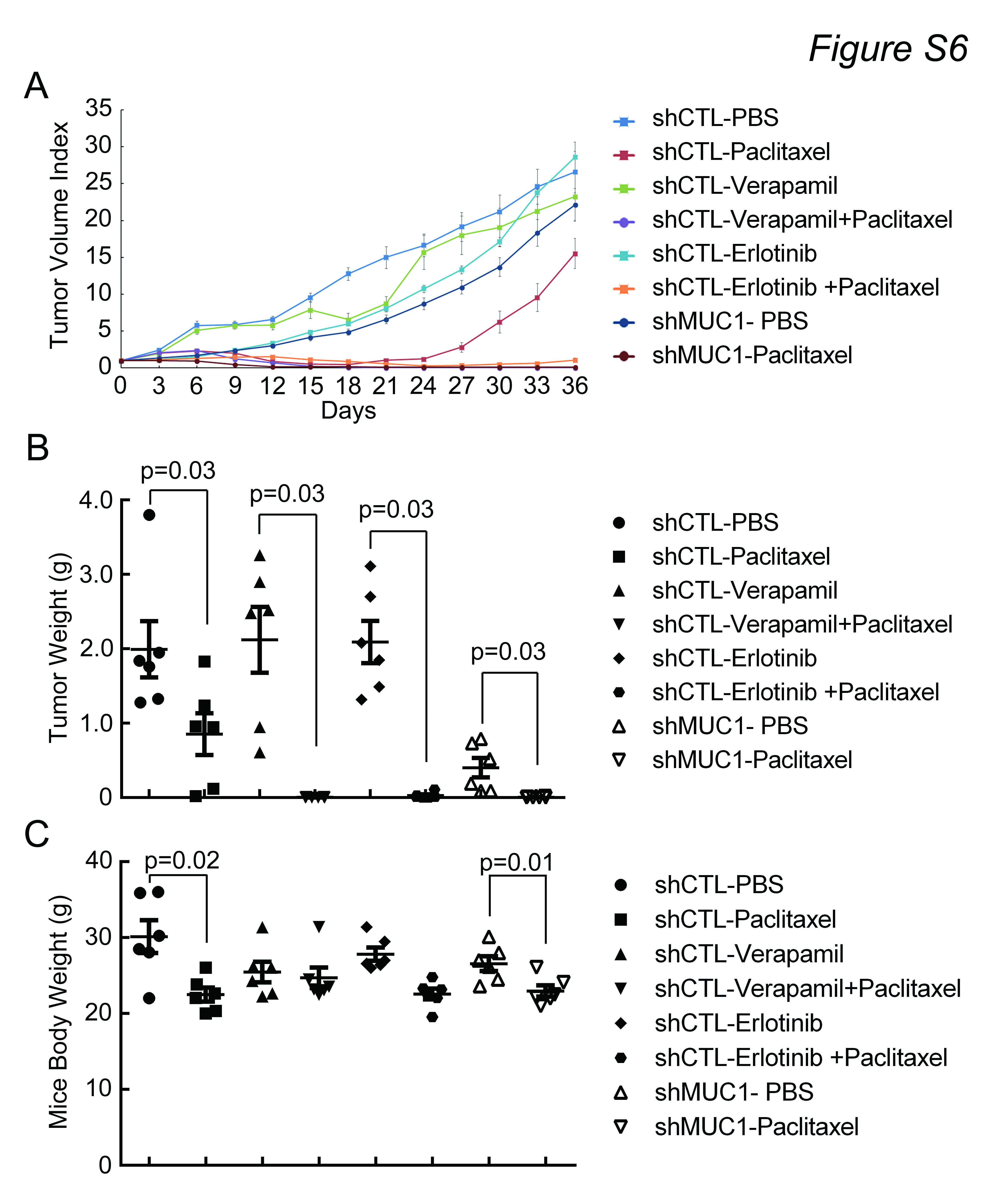
**

**
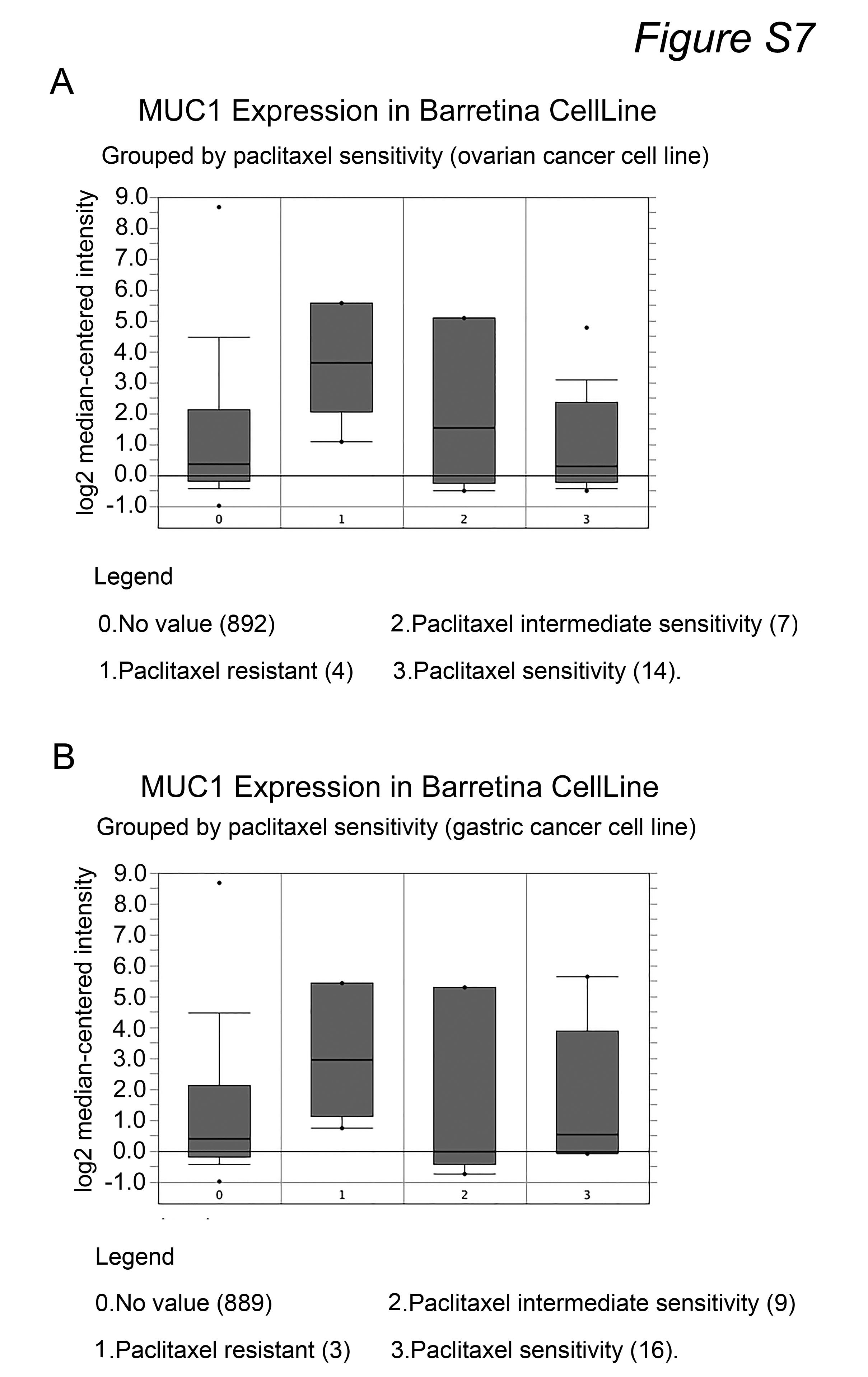
**
